# Sustaining modified behaviours learnt in a diabetes prevention program in regional Australia: the role of social context

**DOI:** 10.1186/1472-6963-12-460

**Published:** 2012-12-17

**Authors:** Christine Walker, Andrea Hernan, Prasuna Reddy, James A Dunbar

**Affiliations:** 1 Chronic Illness Alliance, Melbourne, VIC, Australia; 2 Greater Green Triangle University Department of Rural Health, Flinders University and Deakin University, Warrnambool, VIC, Australia; 3 School of Medicine and Public Health, University of Newcastle, Callaghan, NSW, Australia

**Keywords:** Diabetes prevention, Evaluation, Social context, Self-efficacy, Volition, Qualitative method

## Abstract

**Background:**

The Greater Green Triangle diabetes prevention program was conducted in primary health care setting of Victoria and South Australia in 2004–2006. This program demonstrated significant reductions in diabetes risk factors which were largely sustained at 18 month follow-up. The theoretical model utilised in this program achieved its outcomes through improvements in coping self-efficacy and planning. Previous evaluations have concentrated on the behavioural components of the intervention. Other variables external to the main research design may have contributed to the success factors but have yet to be identified. The objective of this evaluation was to identify the extent to which participants in a diabetes prevention program sustained lifestyle changes several years after completing the program and to identify contextual factors that contributed to sustaining changes.

**Methods:**

A qualitative evaluation was conducted. Five focus groups were held with people who had completed a diabetes prevention program, several years later to assess the degree to which they had sustained program strategies and to identify contributing factors.

**Results:**

Participants value the recruitment strategy. Involvement in their own risk assessment was a strong motivator. Learning new skills gave participants a sense of empowerment. Receiving regular pathology reports was a means of self-assessment and a motivator to continue. Strong family and community support contributed to personal motivation and sustained practice.

**Conclusions:**

Family and local community supports constitute the contextual variables reported to contribute to sustained motivation after the program was completed. Behaviour modification programs can incorporate strategies to ensure these factors are recognised and if necessary, strengthened at the local level.

## Background

Several randomised controlled trials have demonstrated that the onset of type 2 diabetes can be prevented or delayed by lifestyle modification programs [1-3]. Building on this body of evidence to translate small scale successes into ‘real-world’ practice, the Greater Green Triangle implemented a diabetes prevention program (GGT DPP) in the primary health care setting of South-West Victoria and South East South Australia in 2004–2006 [4,5]. The GGT DPP provided evidence of the feasibility of a group-based program and showed an average of 2.5 kg reduction in weight and 4.2 cm reduction in waist circumference at 12 months [4]. In addition, further quantitative evaluation undertaken during the implementation period of the GGT DPP demonstrated significant reductions in risk factors for those who completed the program which were largely sustained at the18 month follow-up [6].

This goal orientated facilitated group lifestyle program was underpinned by social cognitive and self-regulation theories of health behaviour change [7-10]. According to the theories, health behaviour change proceeds from intention information to action initiation and maintenance in two phases: motivation and volition [8,11]. During the motivational phase the major determinants contributing to people's intentions to change their behaviour are risk perception, outcome expectancies, and self-efficacy in decision-making [11,12]. The post-intentional volitional phase can be divided into planning and action including both initiation and maintenance [11]. During the volitional phase, intentions need to be transformed into detailed individual plans about how to perform the desired action [13]. This action process is strongly influenced by perceived self-efficacy, but also by various kinds of self-regulation skills and behaviours e.g., goal setting and planning [8,12,14].

As a functional construct, perceived self-efficacy can be viewed in a phase-specific manner to characterise different functions [15]. Action self-efficacy makes a difference in the pre-action phase and coping self-efficacy reflects beliefs about one's capability to deal with barriers during the maintenance phase.

Appropriate self regulatory strategies are needed to transform intentions into targeted behaviour [14]. One strategy, planning can be divided into two sub-constructs. Action planning specifies intended action in terms of when, where and how to act [7,16]. Coping planning prepares a person for potential obstacles or barriers [16,17]. Following these theories, the GGT DPP intervention was developed to improve participants process of lifestyle change following these theories [18].

The aim of this paper is to investigate how the process of change from psychosocial determinant change to behaviour change and clinical outcomes is perceived from the viewpoint of participants and how other unidentified variables, perhaps exogenous to the research design, may have played a part in the success factors.

As an intervention the GGT DPP is complex because it takes place in a social setting. Further evaluation offers the opportunity to identify whether other factors contribute to the program’s success when it is embedded into routine practices [19,20]. Carl May suggests qualitative evaluations have the advantage of identifying the conditions that will promote or inhibit the introduction of complex interventions into daily practice [21]. Greenhalgh and Swinglehurst in discussing the value of qualitative research methods, such as ethnography in health services research, suggest they provide the ability to study an intervention in the context of the social situation, bringing an understanding of micro-level phenomena such as the local understandings and actions that are an essential though often unacknowledged part of an intervention [22]. Evaluations such as this one contribute to our understanding of why interventions did or did not work and are an important part of the ‘normalisation’ process which takes place in complex local contexts [23]:

"Complex interventions in health care, whether therapeutic or preventative, comprise a number of separate elements which seem essential to the proper functioning of the interventions although the 'active ingredient' of the intervention that is effective is difficult to specify. (…) Complex interventions are built up from a number of components, which may act both independently and interdependently. The components usually include behaviors, parameters of behaviors (e.g. frequency, timing), and methods of organizing and delivering those behaviors (e.g. type(s) of practitioner, setting and location)"

Medical Research Council quoted in May et al. 2007:2 [21].

In line with the work of May [21] and May et al. [23], and Penn et al. [24] we undertook a qualitative evaluation going beyond the program itself to examine context [25]. In November 2009, three to five years after participants completed the GGT DPP the evaluation aimed to explore what social factors beyond the ‘intention to change-behaviour change’ continuum contributed to the success of this intervention in a specific locale and culture. For our purposes the social context included types of practitioner, settings and locations, the broader community and relationships.

## Methods

### Rationale

Qualitative evaluation has the advantage of generating detailed process data from which variables perhaps previously isolated by the quantitative research design but which did play a role in the success of a program may be identified [21,26].

### Design

A focus group methodology [27] was considered the most appropriate qualitative method to generate this level of data from people who would need to recall the program content and their response to that content from several years earlier as well as relating their current activities.

### Setting and participants

Five focus groups were held across the three regional centres in rural Australia where the GGT DPP was originally held: two with men; two with women and one a group comprising both men and women. Participants who had completed the GGT DPP (attended both baseline and 12 month testing sessions) were randomly selected from the three sites the intervention took place according to gender. A total of 116 men and women who completed the GGT DPP were randomly phoned and invited to participate in the focus groups. Recruitment phone calls were placed until 6 – 10 people agreed to participate. Fifty participants agreed to take part in five focus groups, but the final number of people who participated was 29. These 29 participants were generally representative of the total intervention group (n = 237), except focus group participants were significantly older (*p* = 0.031, n = 28), had greater reduction in systolic (*p* = 0.024, n = 28) and diastolic blood pressure (*p* = 0.02, n = 28) over 12 months and had increased physical functioning (p = 0.03, n = 28) as measured by the SF-36 questionnaire over 12 months.

Recruiting from the group that had successfully completed the program meant it was possible to elicit information on those factors that had led individuals from motivation to volition as well as those factors that had contributed to or been barriers to sustaining those behaviours.

Separate groups were organised for men and women to promote and enable gendered discussion. They were asked to recall the content of the program and discuss their views of the content. They were asked what strategies from the program they continued to practise and what suggestions they had for future programs.

### Ethics

Ethical approval was received from Flinders University Clinical Research Ethics Committee (reference number: 105/034). All focus group participants received consent forms and plain language statements. No names have been used in the transcripts.

### Data analysis

Each focus group was recorded and then transcribed. Using a ‘grounded theory’ approach the content of the transcriptions was thematically analysed and compared [28,29].

## Results and discussion

### Analytic categories

Factors contributing to participants’ sustaining modified behaviours fall into two categories. One relates to structure of the program: its design and delivery; the other relates to the broader social context. These categories are artificial constructs of the evaluation and useful for analytical purposes. Participants undertook the program and the subsequent focus group evaluation as part of their daily lives, not distinguishing between structure and context. Consequently as analytical categories they may be seen to overlap and be interrelated.

### Structural aspects of the program

Structural aspects of the program, that is its design and delivery, were noted as contributing to the program’s overall success as well as their individual success in sustaining behaviours after the program was completed. These were: recruitment, risk assessment, the learning environment and facilitators, course content and personal commitment.

### Recruitment

Most people were recruited through their local General Practice (GP) clinic, usually by the practice nurse, although some were initially contacted by hospital or community health centre staff. No-one personally found this form of recruitment intrusive though there was agreement that it might have discouraged some people.

### Risk assessment

The use of the Finnish Diabetes Risk Score (FINDRISC) a self administered, eight question survey tool which can be used to identify people in the community at high risk of diabetes was positively received [30,31]. The assessment of personal risks was considered by participants as the greatest contributor to making a personal commitment to the program. For those who had a family history of type 2 diabetes the additional experience of understanding the implications was an added incentive.

### Learning environment

The focus group participants identified flexibility as an important component of the learning environment. They included access issues such as session times and venues as well as presentations in their assessment of the learning environment.

All groups commented on the value of having session times and venues which fitted in with their own commitments. At the same time, being able to attend a different group’s session time when another commitment interfered was greatly appreciated.

When facilitators were flexible about the sessions and encouraged groups to establish their own activities such as walks and a picnic it was greatly appreciated and seen as contributing to the success of the program.

"The girls [nurse facilitators] were good they went along with the suggestions and built on everything. They asked the participants to list the suggestions like going to the gym or going for a walk and then they’d ask who was interested in doing them."

"(Male focus group participant Horsham)"

### Facilitators

The skills of the health professionals facilitating the sessions were important components of the learning environment. While possessing the knowledge on lifestyle modification was the most recognised skill, focus group participants identified that receiving clear explanations; and having personal concerns addressed in a non-judgmental manner were important qualities.

"They didn’t talk down to you; they talked as though they were in the same boat. It was sort of on our level too."

"(Male focus group participant Horsham)"

The group environment provided participants with the ability to learn from one another and to share their insights related to eating habits, emotions and motivations.

### Course content

Within this group learning environment participants kept food diaries and learnt to read food labels as well as receiving advice about food and exercise and discussing the risk factors they faced. Focus group participants considered the course content to be valuable, which meant they actively engaged with it and could reflect on current behaviour and replace it with new behaviour – these are examples of coping self-efficacy and planning in action.

### Personal commitment

Personal commitment was also an important factor. Participants in the focus groups considered their risk assessment including that a family history of diabetes was part of the risk had given them a ‘wake-up call’. Some men had already started to make lifestyle changes before the program started so that participation in the program contributed to greater adherence to an already existing decision. All focus group participants found that being provided with their pathology test results (cholesterol and glucose levels) as part of the program rather than as part of a doctor’s health check enhanced their ability to assess their own progress. Similarly keeping a food diary was an additional self-assessment tool.

"Sometimes you say I don’t eat much cake but when you write it down you see you are eating cake- and then there was the exercise. I thought I did a lot what with running around after the sheep but when you saw it you knew it wasn’t that much. So that self-assessing that was good."

"(Male focus group participant Hamilton)"

In some instances where the GGT DPP coincided with retirement, adopting an exercise regime became part of the retirement option. However some older people with increasing disabilities were finding it difficult to keep up their exercise regimes.

All focus group participants could recall items they had learned during the program such as how to read food labels, how to reduce fat intake, smaller portions and smaller plate sizes, as well as the level of exercise they needed to undertake to lose weight or maintain fitness. Most reported they had lost weight during the GGT DPP. Self-reported perceptions of weight loss were consistent with the clinical results [4].

All continued to employ at least some of the plans they had made during the GGT DPP on a daily basis, most specifically using smaller dinner plates and the relative food portions, reading food labels as well as exercising which included cycling, walking, playing sports and going to gym.

"I still do it today-stand there in the supermarket and read the packets. I was doing it for muesli just before."

"(Male focus group participant Mt Gambier)"

In some instances people talked of extending their knowledge and nutrition or exercise plans since the program. More exercise such as cycling and going to gym programs were in evidence. One man who had not developed diabetes was regularly using a blood glucose monitor.

"Yes and now when I have a blood test I ask them to test the sugar and I have one of those machines where I can test my sugar. I also carry out some tests on myself like I do an activity like doing a reading and then going out and working in the garden and then doing another reading and doing readings with food to see what happens too. That’s something I wouldn’t have done before."

### The role of the broader social context

Focus groups considered that the broader social context which they defined as their family and community relationships, gender issues and changing life circumstances, played a pivotal role in their success of adopting and sustaining changed lifestyle behaviours.

### Family relationships and gender issues

In this rural community gender issues and family relationships were bound up together. Both men and women in the groups identified that support from their partners was necessary to adopting new behaviours successfully.

"One thing about this program is that you cannot simply go on it as an individual because you have to change your lifestyle. It’s difficult to change your own lifestyle if your partner and family don’t want to change theirs."

"(Male focus group participant Mt Gambier)"

Men were specific that wives needed to provide practical support such as preparing smaller meal sizes and cooking different foods. Otherwise it was too hard to fit their new regimes into the family patterns. Women on the other hand saw men as providing moral support for their new regimes rather than needing practical help. Some people saw themselves as role models for other family members which helped them sustain their own behaviours. A few people reported they had met with indifference or opposition from family members which made it far harder to sustain changes.

"One of the problems I had was coming home after the program and seeing that there weren’t any changes to anyone else’s plate. I was trying to do all the right things but their plates were not like mine."

"(Male focus group participant Hamilton)"

Women generally retained the traditional roles of family care and changed the family diet by stealth (cooking healthier foods and smaller meals without explanation) or openly refusing to buy unhealthy food.

"The different method of shopping-you can make subtle changes without the spouse knowing. I am not as good at reading labels as I should be but I think my shopping has changed."

"(Female focus group participant Hamilton)"

### Changing life circumstances

However changing life circumstances produced changed roles in some families. Some men had retired or semi-retired and had taken on the shopping and cooking while their wives continued to work. This gave them the opportunity to put the strategies they had learned into practice and to exercise some control over the family diet. Penn et al. found similar results regarding retirement and changing behaviour patterns in their evaluation of the United Kingdom-based diabetes prevention program [24]. It also meant that new activities such as walking, joining a walking group or riding bicycles either for sport or leisure could be adopted as part of a retirement plan.

### Community relations

Community relations were also identified as contributing to the success of the program. Sharing in the group sessions with other people and forming exercise groups was an important factor. There was a high level of trust and warmth between group members many of whom already knew one another. The focus groups showed clear evidence that GGT DPP participants found the group settings facilitated their learning from one another. Some participants had extended their group involvement by joining walking groups, cycling clubs or gyms.

The facilitators and the GPs were seen by the participants as part of the community. They were known to most of the participants and generally greatly liked and trusted. The fact that the facilitators made great efforts to make the program enjoyable as well as providing the information in a positive manner with clear explanations was greatly appreciated. Participants considered the level of facilitator commitment a key factor in the program’s success.

There was some evidence of ‘slippage’ amongst focus group participants since they had completed the diabetes prevention program. ‘Slippage’ relates to a gradual compromise in their views of what constitutes a healthy diet to fit more comfortably with their everyday lives. For example, participants argued that households needed supplies of biscuits and cakes for visitors, while savoury scones or biscuits and cheese were healthy alternatives to cake for morning tea. These are the traditional social expectations of an Australian rural community and may be one of the more obdurate features of the broader social context. In practice ‘slippage’ also means a reinterpretation of health messages to legitimise a return to more socially acceptable behaviours. Expressions such as ‘a little bit of chicken skin is OK’ and ‘you are allowed some special treats every so often’ are examples of this. They suggest that some features of the traditional social context have greater magnetism over time and pose a threat to sustaining modified behaviours.

**Figure 1:**
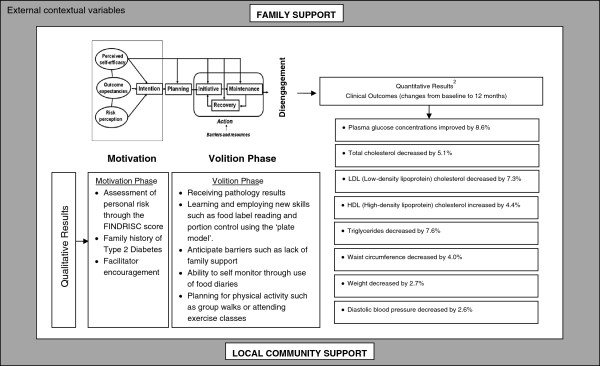
Qualitative findings relation to HAPA model and quantitative outcomes of the GGT DPP.

Schwarzer identified a number of steps in the intention-behaviour change continuum that makes HAPA a successful intervention in a number of settings [9]. These are volitional self-efficacy and strategic planning and the qualitative evaluation demonstrates that the GGT DPP reinforced them. For example, receiving pathology results whereby participants were able to self-monitor their progress reinforced self-efficacy; learning new skills such as label reading had similar effects. People who successfully sustained the program demonstrated a high level of self-efficacy where they were able to anticipate barriers such as lack of family support or a decline in their own motivation. They planned ahead for such barriers. Group walks, cycling for pleasure or sport and attending exercise classes were means to maintain motivation. The GGT DPP introduced strategies so that self-efficacy replaced the initial shock of having a high FINDRISC score. Additionally the convergence between the results of the quantitative evaluation and recall by focus groups participants of their successful weight loss and pathology results suggests a high level of self-efficacy. This self-efficacy has been an important factor in sustaining behaviour changes. Figure 1 illustrates how the qualitative themes uncovered in this evaluation relate to the HAPA model and the quantitative results.

## Conclusions

The GGT DPP is a complex intervention and this evaluation reveals how the psychosocial theories translated into coping self-efficacy and planning by participants, which in turn contributed to clinical outcomes. The evaluation also identified a number of factors external to the intervention itself that played a role in its success. These were family and community relationships.

Participants were strongly aware of the importance of their family relationships to the success of sustained behaviour change. They also identified that gender roles within their largely traditional nuclear family structures were critical elements to success. At the same time, the importance of timing is revealed through the coincidence of the program starting when people’s lives were changing. Retirement is a time for re-assessing one’s life and this meant that the program could be incorporated into changing family roles and freer time. Taking on the new roles of food shopping and meal preparations clearly contributed to gaining greater control over diet and consequently to growing levels of self-efficacy. Moving into retirement meant that plans could be made to incorporate exercise and other changes into available time.

The ‘slippage’ back to previous eating behaviours displayed by some focus group participants demonstrates the importance of the family and community context in sustaining behaviour. The social context of meals which includes family meals, hospitality and display of status in a community influences the consumption of food.

While the community setting where many people already knew one another contributed to successful learning and to the formation of exercise groups at least for the duration of the program, it was the perception that the GGT DPP facilitators and the GPs were part of their own community that was a more important factor. Participants were most appreciative that facilitators gave up their time and shared their knowledge within their community. The importance of family and community relationships in supporting an intervention is underlined by a peer support program that failed when the context in which relationships are established was ignored [32].

It is at this point that the external social factors of family and community can be seen to converge or are intertwined with the intervention itself. Facilitators who lived in the community were able to offer a flexible learning environment. This level of flexibility that was so highly commended by the focus group participants echoes May et al’s view of the ‘normalisation’ process which identified that patients and clinicians need to work together to ‘flexibly configure practices in ways that meet specific *local* situations and requirements’ [23].

Practice nurses who undertook the FINDRISC with potential participants were able to offer a means to address the issue within a community context which captured the FINDRISC as a motivator to move participants into self-efficacy rather than a negative factor. Where changing life circumstances such as retirement converged with the program lifestyle behaviours could be built in and sustained.

Finally, the Diabetes Prevention Program in the Greater Green Triangle was not undertaken as a randomised controlled trial but was an implementation of a complex intervention with people at high risk of developing diabetes in a number of rural communities in Australia [33]. In this sense the project was a process of ‘normalisation’ which embedded an intervention into routine practice [23]. Normalisation relies on everyday use and everyday behaviours of those who participate, rather than innovative leaders or change champions. Whereas May et al. were most concerned with the integration of new programs into everyday clinical settings [23] the GGT DPP was more ambitious in that it integrated lifestyle changes into several community settings. It requires a level of flexibility that takes into account the local setting which in this case includes family and community relationships. It would appear that even at this broad community level, when normalisation successfully takes place lifestyle changes are better sustained.

## Competing interests

The authors have no competing interests to declare.

## Authors’ contributions

JD was the chief investigator of the GGT DPP and was responsible for its design and for obtaining funding. JD and PR were responsible for the research question for this study. CW and AH were responsible for acquisition of data and interpretation of data. CW primarily undertook the analysis. CW wrote the first draft of this manuscript. CW and AH were responsible for its revisions, with JD and PR contributing to specific sections of the manuscript. All authors read and approved the final version of the manuscript.

## Pre-publication history

The pre-publication history for this paper can be accessed here:

http://www.biomedcentral.com/1472-6963/12/460/prepub
